# Oropouche Virus (OROV): Expanding Threats, Shifting Patterns, and the Urgent Need for Collaborative Research in Latin America

**DOI:** 10.3390/v17030353

**Published:** 2025-02-28

**Authors:** André Ricardo Ribas Freitas, David A. Schwartz, Antônio Silva Lima Neto, Rosana Rodrigues, Luciano Pamplona Goes Cavalcanti, Pedro María Alarcón-Elbal

**Affiliations:** 1 Faculdade São Leopoldo Mandic, Campinas 13045, SP, Brazil; romarana.rm@gmail.com; 2 Perinatal Pathology Consulting, Atlanta, GA 30342, USA; davidalanschwartz@gmail.com; 3 Centro de Ciências da Saúde, Universidade de Fortaleza, Fortaleza 60811, CE, Brazil; tanta26@yahoo.com; 4 Faculdade de Medicina, Centro Universitário Christus, Fortaleza 60160, CE, Brazil; pamplona.luciano@gmail.com; 5 Department of Animal Production and Health, Public Veterinary Health and Food Science and Technology, Faculty of Veterinary Medicine, Universidad Cardenal Herrera-CEU, CEU Universities, Calle Tirant lo Blanc 7, Alfara del Patriarca, 45115 Valencia, Spain; pedro.alarconelbal@uchceu.es

**Keywords:** arbovirus, Oropouche virus, emerging zoonotic disease, bunyavirus, congenital infection, vertical transmission

## Abstract

Recent outbreaks of Oropouche virus (OROV) in Latin America demonstrate shifting epidemiological trends, with increasing clinical severity and geographic expansion driven by environmental and anthropogenic factors, many of which remain uncertain. Viral evolution with new reassortant strains, changes in vectors, environmental degradation, and human activities have been postulated as factors that have facilitated its spread into new areas beyond the Amazon Basin. Multiple reports starting in July 2024 of pregnant women with Oropouche fever developing vertical infections and adverse perinatal outcomes, including placental infection, stillbirth, and fetal infections with microcephaly and malformation syndromes, have reinforced the public health significance of this disease. Here, we describe the evidence surrounding this re-emerging epidemic threat, examine these changes, and propose specific strategies for enhanced surveillance and a public health response.

## 1. Expanding Frontiers of Oropouche Virus

Oropouche virus (OROV), an arbovirus of the *Orthobunyavirus* genus, historically caused localized outbreaks in tropical forest regions of South and Central America, primarily in the Brazilian Amazon. Since late 2023, OROV has spread to new territories, including the Caribbean and non-endemic areas of Brazil and Central America, likely driven by new recombinant lineages of the virus [1,2,3]. As of 13 December 2024, there have been 13,014 confirmed cases of Oropouche fever in the Americas region that include imported infections in the United States of America and Canada [4]. For the first time, adult deaths have occurred from Oropouche fever [5].

Traditionally presenting as a self-limiting disease, recent outbreaks have revealed more severe outcomes, prompting investigations into the roles of viral evolution, environmental changes, heightened surveillance, and human activities in shaping its clinical and epidemiological profile. Table 1 summarizes the main evidence currently available on the epidemiological changes and contributing factors associated with this expansion, as well as the knowledge gaps that still need to be addressed.

## 2. Virological Aspects

The recent reemergence of OROV in new geographical areas has been attributed to a monophyletic lineage, OROVBR-2015–2024, which arose through genetic reassortment involving distinct clades [1] and rapidly replaced earlier lineages in the Amazon. Laboratory in vitro studies demonstrated that the OROVBR-2015–2024 strain exhibits higher replication efficiency in mammalian cell cultures compared to the prototype BeAn19991 strain. Additionally, this strain formed more plaques with significantly larger diameters [6]. Plaque reduction neutralization tests conducted with human serum samples from confirmed infections (collected in 2016 in Amazonas, Brazil) and experimentally infected mice revealed that OROVBR-2015–2024 is serologically distinct and significantly less neutralized by antibodies generated from previous strains, such as BeAn19991 [6]. This divergence raises concerns about the possibility of reinfections, emphasizing the need for further studies to clarify the virological implications of this lineage. 

The higher replicative capacity observed in vitro provides critical insights into the competitive advantage of the OROVBR-2015–2024 reassortant strain, potentially indicating an increased ability to infect vectors and enhance replication efficiency that likely contribute to greater infectivity and transmission capacity through both traditional and novel vectors [7]. Coupled with the immunological changes observed in vitro, these factors may help explain the rapid replacement of earlier lineages by OROVBR-2015–2024 in the Amazon region and its exclusive detection in cases of extra-Amazonian transmission during the current outbreak.

## 3. Impact of Climate Change and Human Activities on OROV Spread and Vector Populations

The combined effects of climate change and human activities have played a pivotal role in the expansion OROV outbreaks beyond the Amazon Basin, altering vector ecology and increasing transmission potential in extra-Amazonian regions [8].

Local climate change profoundly affects the life cycle and geographic distribution of OROV vectors, particularly *Culicoides paraensis* (Diptera: Ceratopogonidae). Rising temperatures and extended rainy seasons create optimal conditions for vector reproduction [9]. In extra-Amazonian regions, shifting climatic patterns are fostering the establishment of vector populations previously confined to tropical ecosystems, thereby expanding the potential range of OROV transmission [8].

Climate change and human activities, which have been shown to play crucial roles in the epidemiology of many arthropod-borne viruses, such as deforestation, agriculture, and urban expansion [16], appear to have significantly altered the natural behavior of OROV and driven its geographic expansion beyond its historical pattern of self-limited outbreaks in isolated Amazon Basin villages [1,8]. Deforestation disrupts natural habitats, bringing humans into closer contact with wildlife reservoirs and vectors. In regions like AMACRO (an acronym for the area encompassing parts of Amazonas, Acre, and Rondônia), agricultural-driven deforestation has been linked to notable increases in vector populations along forest edges [9]. The changing infrastructure has not only increased human mobility but also facilitated the transportation of infected vectors and reservoir hosts [9]. This interconnectedness has led to viral dispersal across previously non-endemic regions, including urban and semi-urban centers [8].

## 4. Geographic and Epidemiological Spread

The ongoing OROV outbreak highlights a significant geographic expansion, moving beyond its historically endemic Amazon Basin regions to previously unaffected areas in South and Central America including the Caribbean, and particularly Cuba [2,4]. Cases have been reported in non-endemic regions such as the Atlantic Forest, demonstrating transmission in environments far removed from the virus’s typical tropical rainforest habitat. Increasingly, OROV transmission has been observed in semi-urban and peri-urban zones, where novel ecological conditions and vector species may play a role [9]. However, these transmission events appear to remain largely restricted to vegetated peripheral urban areas, raising important questions about the potential of urban vectors, such as *Culex* mosquitoes, to sustain effective transmission cycles or whether ecological barriers limit their involvement in the virus’s spread [8].

The simultaneous detection of OROV transmission in multiple non-contiguous locations, such as different regions in Brazil and Cuba, suggests a significant epidemiological shift, highlighting the impact of long-distance dispersal caused by humans [9]. Phylodynamic reconstructions revealed that 22% of the current spread of OROV within the Amazon region involves long-range migrations (>10 km), consistent with viral dispersal facilitated by human activity. Factors such as migration, trade, tourism, and transportation networks are likely driving this movement, allowing the virus to establish itself in geographically distant and ecologically diverse regions [1]. These findings highlight the key role of human behavior in shaping the geographic expansion of OROV, challenging traditional assumptions about its transmission dynamics and raising urgent concerns about its potential for infiltration into urban areas and crossing of international borders [2].

The detection of OROV transmission in regions with high tourist traffic, particularly in countries in the northern hemisphere, increases the risk of introduction of this vector-borne disease into new territories that share similar climatic seasons. This potential for global spread highlights the critical need for proactive surveillance in non-endemic areas.

## 5. Transmission and Vector-Host Dynamics

By November 2024, only seven studies had examined vector competence for OROV. The virus is primarily transmitted to humans by *C. paraensis*, a biting midge widely distributed in the Americas, from the United States to Argentina, known for thriving in anthropogenic environments and exhibiting generalist feeding behavior targeting humans and domestic animals [10]. Another potential vector is *Culicoides sonorensis*, which has shown limited OROV transmission capacity under experimental conditions. The proximity of vector breeding sites to human habitation is a significant risk factor for OROV infection, influenced by the anthropophilic behavior of these insects. In this regard, a good candidate could be *Culicoides insignis*, which spreads from the southeastern Unites States through Central America, the Caribbean, and South America, where it is considered an important vector of bluetongue virus, a disease that affects sheep, cattle, and other ruminants. In fact, a recent study confirmed this widespread biting midge species as a potential OROV vector in the Peruvian Amazon [17]. Other anthropophilic *Culicoides* species may also contribute to OROV transmission; however, studies examining their activity patterns, vectorial capacity, and competence are needed to confirm this potential [18].

Mosquitoes (Diptera: Culicidae) are suspected of playing a minor role in OROV transmission, with species like *Coquillettidia venezuelensis* and *Aedes serratus* proposed as sylvatic vectors, and *Culex quinquefasciatus* as a potential urban vector. However, mosquitoes (including both *Aedes* and *Culex* spp.) exhibited an infection rate consistently below 20%, and showed limited OROV transmission [10].

Notably, neither *C. paraensis* nor *C. sonorensis* has been documented to date in the Greater Antilles (which does not rule out their presence), where OROV was first reported in Haiti a decade ago [12]. Emerging scenarios, such as the current epidemic in Cuba, have led to speculation about a potential shift toward mosquito vectors; nonetheless, no scientific evidence currently supports this hypothesis. In fact, biting midges remain significantly under-researched compared to other arthropod vectors in the Caribbean, such as mosquitoes, sandflies, and ticks [19].

This arbovirus is believed to be maintained in a sylvatic cycle, with wild animals acting as reservoirs, including sloths, nonhuman primates, rodents, and birds [20]. Additionally, reports of OROV infections in individuals living far from forested areas suggest the potential existence of an urban transmission cycle. The potential role of domestic animals has recently been suspected in Brazil as neutralizing antibodies to OROV have been detected in cattle and dogs [13]. During previous outbreaks, domestic birds such as chickens and ducks were shown to have antibodies against OROV, and consequently they have been suggested as amplifiers [20]. However, the existing literature on OROV detection in reservoir hosts is limited, resulting in a lack of scientific evidence to support this hypothesis.

## 6. Clinical Impacts

Since March 2024, severe reported cases of OROV infection have revealed significant changes in its clinical profile [2]. In Bahia, two previously healthy women aged 21 and 24 with no risk factors succumbed to rapid disease progression characterized by hemorrhagic manifestations and multi-organ dysfunction [5]—the first deaths attributable to OROV infection. In Pernambuco, a 28-year-old pregnant woman at 30 weeks of gestation developed acute febrile illness, which culminated in fetal demise; tests confirmed OROV RNA in the placenta and fetal organs [2]. Additionally, a well-documented and peer-reviewed published case from Ceará provided robust evidence of vertical transmission. This case involved a 40-year-old pregnant woman in her third trimester who developed an acute febrile illness diagnosed as OROV [11]. Following a stillbirth, OROV RNA was detected in umbilical cord blood and various fetal tissues including the placenta, lung, heart, liver, spleen, and central nervous system. Maternal and fetal viral sequences showed close similarity [11]. Alternative infectious causes associated with fetal loss or acute hemorrhagic febrile syndromes, such as dengue, Zika, chikungunya, and yellow fever, were rigorously excluded in all cases. Other retrospective studies have suggested OROV to be a potential cause of congenital malformations, including microcephaly, with findings of OROV IgM in newborns and maternal samples, and OROV RNA and antigens detected in multiple fetal tissues in severe cases [3,11,14,15,21,22]. While the mechanisms of intrauterine OROV transmission remain unknown, they are likely the result of maternal viremia followed by binding of OROV to as-of-yet unknown surface receptors on placental cells at the maternal–fetal interface, placental infection, and viral passage into the fetal blood stream [15,21].

Another new manifestation of the novel reassortant strains is its ability to infect semen. Replication-competent OROV has been identified in the semen of a 42-year-old healthy man who developed Oropouche fever while visiting Cuba [23], a finding that introduces the potential for additional complications of Oropouche fever including sexual transmission.

The emergence of severe forms, previously unreported in adults, and vertical transmission with adverse fetal outcomes linked to the OROV-2023-24 lineage are suggestive of changes in pathogenicity [5,11]. These observations align with in vitro findings of increased viral replication in mammalian cells and altered responses to pre-existing antibodies [6]. However, it is premature to draw definitive conclusions. Additional studies on pathogenicity and virus–host interactions are essential, as these findings could also reflect the higher absolute number of cases and improved diagnostic capacity for detecting severe clinical presentations.

## 7. Concluding Remarks and Implications for Public Health

The remarkable and rapid spread of OROV may be attributed to a combination of interrelated factors. Genetic evolution of the virus has occurred against a backdrop of significant environmental degradation, characterized by unprecedented deforestation, illegal mining, and the human occupation of vast areas of the Brazilian Amazon. These activities have brought the virus closer to densely populated regions, facilitating its transmission. This gradual increase in infections has created a large pool of viremic individuals, amplifying the potential for further spread.

Compounding these factors is the high mobility of individuals exploring the Amazon and the existence of favorable ecological conditions for vector presence outside the Amazon Basin. Studies during the ongoing outbreak revealed that Oropouche fever cases were concentrated in rural areas of small municipalities with agriculture-modified environments [9]. Notably, transmission was significantly higher in villages surrounded by banana plantations and cassava cultivation. Furthermore, *C. paraensis*, the primary vector of OROV, has dispersed across various countries, adapting to diverse environmental and ecological conditions. It is, therefore, necessary that entomological studies focusing on biting midges, which combine traditional morphological identification methods with new molecular techniques, as well as studies on vector competence and capacity, be promoted, especially in those countries with significant knowledge gaps regarding these telmophagous insects [24].

Public awareness campaigns to educate communities about biting midges and other potential vector species, and targeted interventions to control their populations, are equally vital to reduce transmission risks. As OROV continues to extend its reach into non-endemic regions, fostering regional and international collaboration will be essential to mitigate its spread and protect vulnerable populations. These efforts must be supported by robust research, policy development, and resource allocation to ensure comprehensive preparedness and response to this re-emerging arboviral threat.

## Figures and Tables

**Table 1:** Comprehensive overview of key factors in the current Oropouche virus outbreak.

Major Category	Current OROV Outbreak Situation	Supporting Evidence	Contradictory Evidence	Gaps	Sources
**Virological aspects**	**OROVBR-2015–2024 clade as responsible for epidemiological shifts**	Increased incidence potentially linked to unique genetic characteristics of the OROVBR-2015–2024 clade	May be attributed to improved virological surveillance and environmental factors	Insufficient longitudinal clinical and genetic studies on different clades	[1,6,7]
**Climate Change and Human Activities**	**Climate change and human activities have facilitated the expansion of OROV outbreaks beyond the Amazon Basin**	Shifting climate patterns could create optimal conditions for Culicoides paraensis reproduction, enabling vector populations to establish in previously unaffected areas	Limited data on vector ecology in extra-Amazonian regions	Lack of integrated surveillance systems to assess the interaction of environmental drivers and vector dynamics	[1,8,9]
	**Deforestation, infrastructure changes facilitate and urban expansion increase human-vector contact vector movement and viral dispersal.**	The worst epidemic in the Amazon region has been observed in AMACRO, where agricultural expansion and the construction of roads, such as the BR-319 highway, may have facilitated the movement of infected vectors and reservoir hosts	Limited empirical data directly links infrastructure projects to OROV outbreaks, as other environmental or social factors may also contribute to viral spread	There is a lack of comprehensive studies investigating the specific mechanisms through which deforestation and infrastructure changes, such as roads, alter vector ecology and drive viral dispersal	[1,8,9]
**Geographical and Epidemiological Spread**	**Territorial expansion**	Occurrence in new locations across South and Central America, including the Caribbean	None	Limited surveillance data in remote or sparsely populated regions	[1]
	**Human-Mediated Introduction to New Regions**	Documented transmission in areas non-contiguous with the Amazon	None	Insufficient virological surveillance of human and zoonotic cases outside endemic zones	[1]
	**Potential Spread to Urban Centers**	Reported cases in urban areas, indicating potential adaptation to urban vectors	Confirmed transmission limited to peripheral urban areas with vegetation, not central urban environments	Inadequate vector control and monitoring in central urban settings to confirm or refute urban transmission	[10]
**Transmission and Vector-Host Dynamics**	**Sustained Transmission in New Biomes**	Documented cases in regions with diverse vegetation and fauna, suggesting adaptation	Outbreaks in these locations continue to predominantly coincide with dense vegetation zones	Lack of detailed ecological studies to map transmission patterns across varied ecosystems	[5,6,7,11]
	**Emergence of New Vector Species**	Detection in locations with diverse fauna, suggesting adaptation to additional vector species	No successful isolation of OROV in new suspected vectors	Need for experimental studies on vector competence in novel species and strains	[7,10,12]
	**Increased Capacity to Infect Known Vectors**	Increased replicative efficiency and higher viral loads in mammalian cells suggesting enhanced infection potential	No consistent evidence confirming increased transmission efficiency in recognized vector species	Lack of studies examining vector competence and transmission dynamics in both traditional and potential new vectors	[6,10]
	**Potential Role of Domestic Zoonotic Reservoirs**	Cases detected without proximity to sylvatic areas, suggesting possible role of domestic animals	Role of domestic animals in sustaining transmission remains unclear	Lack of longitudinal studies to confirm reservoir role in domestic animals and new local fauna	[10,13]
**Clinical Impacts and Immune Challenges**	**Increased Severity and Novel Clinical Presentations**	Previously unreported fatalities and high replication rates in mammalian cells	Increased severity may reflect higher case numbers rather than an actual shift in the clinical profile	Need for detailed clinical characterization and in vivo studies to assess severity trends to determine if observed severity represents a genuine clinical shift or is due to reporting biases.	[5,11,14,15]
	**Potential Immune Escape in Previously Infected Individuals**	Neutralizing antibodies from previous OROV infections show low efficacy against OROV-2023–24	Cross-reactivity in some serological tests has not been observed consistently	Need for clinical and laboratory studies to fully evaluate immune response and confirm potential immune escape mechanisms	[6]
	**Vertical Transmission and Fetal Impacts**	Reports of congenital abnormalities and miscarriages among infected pregnant individuals	No large-scale systematic data confirming consistent vertical transmission	Insufficient data collection on pregnancy outcomes and impacts of OROV infection in pregnant women	[11,14]
